# Birth Outcomes of Newborns after Folic Acid Supplementation in Pregnant Women with Early and Late Pre-Eclampsia: A Population-Based Study

**DOI:** 10.4061/2011/127369

**Published:** 2010-11-30

**Authors:** Ferenc Bánhidy, Abdallah Dakhlaoui, István Dudás, Andrew E. Czeizel

**Affiliations:** ^1^Second Department of Obstetrics and Gynecology, School of Medicine, Semmelweis University, Budapest, Hungary; ^2^Department of Pulmonology, Elisabeth Teaching Hospital, Sopron, Hungary; ^3^Foundation for the Community Control of Hereditary Diseases, Törökvész lejtö 32, 1028 Budapest, Hungary

## Abstract

*Objective*. To evaluate the rate of preterm birth and low birth weight in the newborns of pregnant women with early and late onset pre-eclampsia according to folic acid supplementation. *Study design*. Birth outcomes of newborns were evaluated in 1,017 (2.7%) pregnant women with medically recorded pre-eclampsia and 37,134 pregnant women without pre-eclampsia as reference in the Hungarian Case-Control Surveillance System of Congenital Abnormalities, 1980–1996, in addition these study groups were differentiated according to the supplementation of high dose of folic acid alone from early pregnancy. *Results*. Pregnant women with pre-eclampsia associated with a higher rate of preterm birth (10.2% versus 9.1%) and low birthweight (7.9% versus 5.6%). There was a lower risk of preterm birth (6.8%) of newborn infants born to pregnant women with early onset pre-eclampsia after folic acid supplementation from early pregnancy though the rate of low birthweight was not reduced significantly. There was no significant reduction in the rate of preterm birth and low birthweight in pregnant women with late onset pre-eclampsia after folic acid supplementation. *Conclusion*. The rate of preterm birth in pregnant women with early onset pre-eclampsia was reduced moderately by high doses of folic acid supplementation from early pregnancy.

## 1. Introduction

Pre-eclampsia (PE) is frequent (2–8%) and severe complications of pregnancy, and this multisystem disorder of pregnancy is characterized by pregnancy-induced hypertension and new-onset proteinuria during the second half of pregnancy [1–3]. PE is a major contributor to maternal mortality if associates with eclampsia and HELLP syndrome [4, 5]. Furthermore, since delivery is the only cure of PE, there is a higher risk of preterm birth up to 15% [6] and intrauterine growth retardation [7] with an increase in infant mortality and morbidity.

Two important hypotheses have been generated for the pathogenesis of PE during the last decades. The first hypothesis was based on the differentiation of early and late onset PE [3] or on the two-stage model of PE [8]. The second hypothesis was based on PE associated with placental insufficiency due to hyperhomocysteinemia-related vasculopathy because 3.2–7.7-fold higher risk of PE was found in pregnant women with elevated homocysteine levels [9–16]. Folic acid supplementation lowers plasma homocysteine in general [17] and in patients with PE [18], thus folic acid containing multivitamins was tested in pregnant women with gestational hypertension [19] and in pregnant women with PE [20, 21] with significant preventive effect. However, the effect of folic acid alone was not tested though most pregnant women use only folic acid.

The first objective of our study was to evaluate the birth outcomes of pregnant women with early and late onset PE, while the second aim was to check the effect of folic acid supplementation for the risk of preterm birth and low birth weight in their newborn infants in the population-based data set of the Hungarian Case-Control Surveillance of Congenital Abnormalities (HCCSCA) [22].

## 2. Material and Methods

The HCCSCA is based on the comparison of exposures studied during the pregnancy of mothers of cases with different congenital abnormalities and the mothers of controls without any defect matched to the cases. Cases with congenital abnormalities are selected from the Hungarian Congenital Abnormality Registry [23] for the HCCSCA. Control newborns were selected from the National Birth Registry of the Central Statistical Office for the HCCSCA. In general, two newborns were matched individually to each case according to sex, week of birth in the year when cases were born, and district of parents' residence of cases. 

Cases were excluded from this analysis because congenital abnormalities may have a more robust effect for birth outcomes than PE. Thus only 38,151 control newborns without any defect of the HCCSCA, 1980–1996, were evaluated in this study. 

Immediately after the selection of newborns an explanatory letter was sent to the mothers, and they were asked to send us the prenatal maternity logbook and all other medical records regarding the study pregnancy; these documents were sent back after three weeks. Prenatal care was mandatory for pregnant women in Hungary (if somebody did not visit prenatal care clinic, she did not receive a maternity grant and leave), thus nearly 100% of pregnant women visited prenatal care clinics, an average 7 times in their pregnancies. The first visit was between the 6th and 12th gestational week. The task of obstetricians was to record all pregnancy complications, including PE, maternal diseases, and related drug prescriptions in the prenatal maternity logbook. 

On the other hand, a structured questionnaire, a list of medicines (drugs and pregnancy supplements), and a printed informed consent form were also mailed to the mothers. The questionnaire requested information on maternal personal (e.g., employment status) and medical data including pregnancy complications, maternal diseases, and medicine intakes during the study pregnancy according to gestational month. In order to standardize the answers, mothers were asked to read the enclosed lists as a memory aid before they replied and to send back the filled-in questionnaire and informed consent form with their signature in our prepaid envelop.

The interval between the end of pregnancy and return of the “information package” including prenatal maternity logbook, questionnaire, and so forth, was 5.2 + 2.9 months. 

In addition, 200 nonrespondent and 600 respondent mothers were visited at home by regional nurses as part of two validation studies [24, 25] because the committee on ethics considered this followup to be disturbing to the parents of all healthy children. Regional nurses helped mothers to fill in the questionnaire used in the HCCSCA, evaluated the available medical documents, and obtained data regarding the lifestyle of mothers through a cross interview of fathers and other close relatives living together, and finally the so-called family consensus was recorded. 

Overall the necessary information was available on 83.0% of pregnant women (81.3% from reply and 1.7% from home visit). Here the 17 years' data of the HCCSCA between 1980 and 1996 are evaluated because the data collection has been changed since 1997 (all mothers are visited by regional nurses), and the recent data had not been validated at the time of the analysis. 

Four types of hypertension in pregnant women were classified: (i) chronic hypertension, (ii) PE, (iii) PE superimposed upon chronic hypertension, and (iv) gestational hypertension [1, 2, 26]. Our plan was to evaluate the possible association of these four types of hypertension in pregnant women with the risk of other pregnancy complications and adverse birth outcomes. The data of our study regarding chronic and gestational hypertension were published [27], while the evaluation of pregnant women with PE superimposed upon chronic hypertension is in process. Here the birth outcomes of pregnant women with PE are presented.

Blood pressure and proteinuria were measured in pregnant women at their visits in the prenatal care clinics. If a new-onset proteinuria was found by the help of dipstick screening test after the 20th gestational week, pregnant women were referred to a detailed laboratory examination (more than 300 mg in 24 h was accepted as proteinuria). PE was diagnosed in pregnant women if they had new onset hypertension and proteinuria. Of course, pregnant women with secondary hypertension were also excluded from the study. 

The preliminary analysis of data showed that the diagnosis of PE in the questionnaire based on retrospective maternal information was not reliable; therefore, we decided to evaluate only medically recorded PE in the prenatal maternity logbook. There is no consensus in the classification of early and late onset PE, SOGC [28] and ASH [29] recommended for late onset PE less than 34 or 35 gestational week, respectively, while others differentiate early onset before 24 weeks gestation [30]. Thus we decided to evaluate the onset of PE according to gestational months. 

Other pregnancy complications, PE-related drug treatments and other potential confounding factors such as maternal age, birth order, marital and employment status as indicators of socioeconomic status [31], other maternal diseases, and folic acid supplements were also evaluated. 

Only one type of 3 mg folic acid (Alkaloida/ICN Hungary) tablet was available in Hungary during the study period. 

Gestational age was calculated from the first day of the last menstrual period. Both birth weight and gestational age at delivery were medically documented in the discharge summary of mothers because all deliveries took place in inpatient obstetric clinics. The rate of low (less than 2500 gram) and large (4000 gram or more) birth weight, in addition to the rate of preterm (less than 37 completed gestational weeks or less than 259 days) and postterm (42 completed weeks or 294 days or more) birth was calculated. 

### 2.1. Statistical Analysis of Data

 We used SAS version 8.02 (SAS Institute, Cary, North Carolina, USA) for statistical analyses. The occurrence of folic acid use was compared in pregnant women with PE and the *reference group *including all pregnant women without PE. Contingency tables were prepared for the main study variables. First, the characteristics of pregnant women with different study groups were compared with the reference group using chi-square test for categorical variables and Student *t*-test for quantitative variables. Second, frequency of maternal diseases, pregnancy complications, and related drug treatments was compared between mothers with PE and the reference group by ordinary logistic regression models and odds ratios (OR) with their 95% confidence intervals (CI) being evaluated. Third, the birth outcomes of newborns were evaluated in mothers with PE compared with the reference group using adjusted Student *t*-test and OR with 95% CI using ordinary logistic regression model. Finally birth outcomes of newborns in pregnant women with PE were stratified according to folic acid supplementation.

## 3. Results

The total number of births in Hungary was 2,146,574 during the study period, thus 38,151 controls represented 1.8% of all Hungarian births. Of these 38,151 newborns, 1,017 (2.7%) had mothers with medically recorded PE in the prenatal maternity logbook. Of these 1,017 pregnant women with PE, 45 (4.4%) had later eclampsia while HELLP was not recorded. 

Only 580 (57.0%) women out of total 1,017 who were diagnosed as PE had recorded history of taking folic acid supplementation, while this figure was 54.4% (20,195/37,134) in the reference group, thus pregnant women with PE used somewhat more frequently folic acid. The indication of folic acid supplementation was the prevention of neural tube defects. The distribution of daily folic acid supplementation was the following: 22.5%, 68.6%, and 8.9% of pregnant women used one (3 mg), two (6 mg), and three (9 mg) tablets, respectively. Thus the estimated mean daily dose was 5.6 mg. The onset of folic acid supplementation was in about 10% of pregnant women before conception; however, most women started folic acid use after the first visit in the prenatal care clinic, that is, between the 6th and 12th gestational weeks, thus before the onset of PE. Practically all pregnant women continued folic acid supplementation until the end of pregnancy. Of 580 pregnant women with PE and folic acid use, 440 (75.9%) had medically recorded folic acid use in the prenatal maternity logbook while this figure was 61.3% in the reference group. Our validation study showed that maternal information regarding folic acid use was correct, but some women retrospectively forgot to mention it. Folic acid containing multivitamins was used rarely and it contained different low doses of folic acid, thus these pregnant women were excluded from the study. 

Maternal characteristics are shown in Table 1. There was no difference in mean maternal age of pregnant women with or without PE, while mean birth order was lower by 0.3 in pregnant women with PE due to the higher proportion of primiparous pregnant women. In addition, the difference in the mean pregnancy order (birth + miscarriages) was 2-fold higher in women with PE (0.4) than in pregnant women without PE (0.2), and these findings indicate a higher rate of miscarriages in the previous pregnancies of women with PE. The proportion of professional women was somewhat lower in pregnant women with PE, while their proportion of managerial women and skilled workers was somewhat higher compared to pregnant women without PE. Pregnant women with PE and folic acid use were somewhat older with higher mean birth order.

Acute and chronic maternal diseases did not show significant differences between pregnant women with PE and the reference sample. 

Among other pregnancy complications, threatened abortion (20.8% versus 17.0%) and placental disorders (2.2% versus 1.5%), particularly abruption placentae occurred more frequently in pregnant women with PE than in pregnant women without PE.

Practically all pregnant women with PE were treated with antihypertensive drugs, most frequently nifedipine (15.1% versus 2.2%) and methyldopa (10.5% versus 0.9%) compared with pregnant women without PE. Dihydralazine, metoprolol, clopamide, and furosemide were also more frequently used by pregnant women with PE. However, magnesium sulphate was used only in two pregnant women with PE.

The diagnosis of PE according to gestational months is shown in Table 2. These data reflect the record of PE diagnosis in the prenatal maternity logbook, but it may be near to the onset of this pregnancy complication due to the frequent visits in prenatal care clinics. However, of 1,017 pregnant women with PE, 100 (9.8%) had not unambiguous time of diagnoses. Unexpectedly the diagnosis of PE was recorded in the fourth gestational month in 3.9% of pregnant women. In general, these pregnant women had new-onset hypertension but proteinuria was confirmed after the 20th gestational week. The maximum was found during the last two pregnancy months.

The birth outcomes of newborn infants born to pregnant women with PE and without PE as reference are shown in the lower part of Table 2. (There was no significant difference in the sex ratio of the study groups.) The mean gestational age was the same in pregnant women with or without PE but the rate of preterm birth was somewhat but not significantly higher in the group of pregnant women with PE (10.2% versus 9.1%). The mean birth weight of newborn infants born to pregnant women with PE was somewhat (41 g) larger compared to the newborns of pregnant women without PE and this small difference was significant. On the contrary, the rate of low birth weight newborns was higher in the group of pregnant women with PE (7.9% versus 5.6%), and this 40% increase is significant on both statistical and clinical aspects. 

In the next step, newborns were evaluated according to gestational age and birth weight groups in pregnant women with PE and without PE as reference. A characteristic U-shaped curve was shown; the previously mentioned higher rate of preterm birth and low birth weight associated with a higher rate of postterm birth (11.2% versus 10.1%; OR with 95% CI: 1.1. 0.7–1.7) and large birth weight (10.9% versus 7.4%; OR with 95% CI: 1.5, 1.2–2.0). However, these differences reached the level of significance only in low and large birth weight, and the mean birth weight was higher both in term and the postterm births.

The gestational age at delivery and birth weight were moderately modified by folic acid supplementation from the early pregnancy (Table 2). The mean gestational age was 0.3 week longer in pregnant with PE after folic acid supplementation compared to pregnant women with PE, but without folic acid use, These data were in agreement with their lower rate of preterm births (8.8% versus 12.1%). However, folic acid supplement associated with only 46 g larger mean birth weight and with moderate reduction of low birth weight (7.2% versus 8.7%) and these differences were not significant. If only medically recorded folic acid uses were considered, the data of birth outcomes did not differ from the total data.

However, the effect of folic acid increased the rate of postterm birth (11.9% versus 11.2%) with smaller mean birth weight and the rate of large birth weight (11.6% versus 10.9%) with shorter mean gestational age (Table 3), though these changes did not reach the level of significance.

The birth outcomes were evaluated according to the onset of PE (Table 2). There was no obvious trend in mean gestational age and birth weight depending on the onset of PE. The fifth and last ninth months had longer gestational age with the lower rate of preterm birth. The fourth month due to possible diagnostic bias associated with extremely high preterm birth. Obviously the major risk for adverse birth outcomes was connected with the onset of PE between the sixth and eighth gestational months. 

Finally the effect of folic acid for birth outcomes was evaluated in pregnant women with early (IV–VI months) and late (VII–IX months) onset PE in the study (Table 4). Folic acid was able to reduce significantly the rate of preterm birth (OR with 95% CI: 0.41, 0.18–0.94) in pregnant women with early onset PE. There was also a reduction in the rate of low birth weight rate after folic acid use in early pregnancy of women with early onset PE but this reduction did not reach the level of significance (OR with 95% CI: 0.52, 0.19–1.40).

## 4. Discussion

The primary aim of the study was to evaluate birth outcomes of pregnant women with early (“placental”) and late (“maternal”) PE. The risk of adverse birth outcomes cannot be differentiated according to early and late onset PE defined in our study, because the onset between sixth and eighth gestational months associated with a higher risk of preterm birth and low birth weight. The secondary aim of the study was to estimate the possible effect of folic acid for the risk of preterm birth and low birth weight of their newborns. On one hand, the high dose of folic acid used in early pregnancy reduced the rate of preterm birth but not modified significantly the higher rate of low birth weight, that is, intrauterine growth retardation in pregnant women with early PE. On the other hand, this preventive effect of folic acid was not observed in pregnant women with late PE; in fact, there was somewhat but not significantly higher rate of preterm birth and low birth weight in pregnant women with late PE after folic acid supplementation.

The prevalence of PE was 2.7% in pregnant women without PE superimposed upon chronic hypertension. Eclampsia is defined as the occurrence of tonic-clonic seizures in pregnant women with PE, and recently eclampsia has manifested only in 1-2% of women with severe PE [3]. However, this figure was 4.4 % in the study explained by the possible overdiagnoses and mainly by the lack of magnesium sulphate treatment in Hungary during the study period. 

The primary goal is to prevent the manifestation of PE with maternal complications; however, a reasonable secondary goal is to reduce the adverse birth outcomes of pregnant women with PE. The rate of preterm births (10.2% versus 9.1%) and mainly of low birth weight (7.9% versus 5.6%) was higher in the newborns of pregnant women with PE than in the newborns of pregnant women without PE, though these figures were lower than rates found in previous studies [32, 33]. These differences may reflect the improvement of medical care of pregnant women with PE. However, the unexpected findings of the study were the higher rate of postterm birth (11.2% versus 10.1%) and mainly of large birth weight (10.9% versus 7.4%) in the newborns of pregnant women with PE. The latter explains the larger mean birth weight of newborn infants born to pregnant women with PE compared to the newborns of pregnant women without PE. The mild U-shaped distribution of gestational weeks is in agreement with the same mean gestational age at delivery in the groups of pregnant women with or without PE.

The use of folic acid modified these findings; the mean gestational age was somewhat longer with a lower rate of preterm birth. Similar beneficial effect was not found in the rate of low birth weight newborns. However, an important observation of our study is that the beneficial effect of high dose of folic acid use from early pregnancy occurred only in pregnant women with early (placental) onset PE. 

Our previous study showed that the high dose of folic acid during pregnancy associated with a minor increase of birth weight but a longer gestational age at delivery and significant reduction in the rate of preterm birth [34]. In this study, we found similar findings, but these effects were moderate, thus the maternal pathological conditions are important in the effect of folic acid.

The major expected benefit of antihypertensive therapy is the reduction of hypertension of pregnant women with PE [27] to reduce maternal complications and adverse birth outcomes. However, it was not successful in many women in our material. Magnesium sulphate is recommended for the treatment of severe PE [35, 36]; unfortunately, it was used rarely in Hungary.

Our study confirmed the role of nulliparity [3] and the higher rate of previous miscarriages [37] in the origin of PE. 

The strengths of the HCCSCA are that it is a population-based large data set including 1,017 pregnant women with prospectively and medically recorded PE in an ethnically homogeneous European (Caucasian) population. We were able to differentiate PE from the other types of hypertension in pregnant women. Additional strengths are medically recorded other pregnancy complications and birth outcomes, in addition to the available data of potential confounders. 

However, this data set also has limitations. (i) Other pregnancy outcomes, for example, miscarriages were not known in the study pregnancy. (ii) Lifestyle factors could not be evaluated in the total data set due to the unreliability of maternal self-reported data [38], though smoking seems to be protective for PE [39–41].

In conclusion, a higher risk of preterm births and mainly low birth weight was found in the newborns of pregnant women with PE. The use of high dose of folic acid from early pregnancy resulted in a reduction of preterm birth in pregnant women with early onset PE; however, similar beneficial effect was not found in the rate of low birth weight and in pregnant women with late onset PE. The clinical importance of these statistically significant changes is necessary to be validated in well-controlled prospective studies.

## Figures and Tables

**Table 1:** Characteristics of pregnant women without pre-eclampsia (PE) as a reference and with PE, in addition pregnant women with PE supplemented with folic acid.

Variables	Pregnant women				Pregnant women with PE + folic acid	
	without PE (*N* = 37,134)		with PE (*N* = 1,017)		folic acid (*N* = 580)	
Maternal age (yr)	No.	%	No.	%	No.	%

19 or less	3,191	8.6	86	8.5	43	7.4
20–29	26,877	72.4	725	71.3	410	70.7
30 or more	7,066	19.0	206	20.3	127	21.9
Mean ± S.D.	25.5	4.9	25.5	5.0	25.7	4.9

Birth order						
1	17,603	47.4	706	69.4	340	58.6
2 or more	19,529	52-6	311	30.6	240	41.4
Mean ± S.D.	1.7	0.9	1.4	0.9	1.6	1.1

Pregnancy order						
1	15,780	42.5	540	53.1	301	51.9
2 or more	21,354	57.5	477	46.9	279	48.1
Mean ± S.D.	1.9	1.2	1.8	1.2	1.8	1.1

Unmarried	1,443	3.9	29	2.9	14	2.4
Employment status Professional	4,330	11.7	93	9.1	56	9.7
Managerial	9,960	26.8	305	30.0	181	31.2
Skilled worker	1,551	31.1	357	35.1	202	34.8
Semiskilled worker	5,998	16.2	163	16.0	89	15.3
Unskilled worker	2,140	5.8	47	4.6	24	4.1
Housewife	2,310	6.2	40	3.9	22	3.8
Others	841	2.3	12	1.2	6	1.0

**Table 2:** Onset (diagnosis) of pregnant women with pre-eclampsia according to gestational month and their birth outcomes.

Gestational months	No.	%	Gestational age (wk)		Birth weight (g)		Preterm birth		Low birth weight	
			Mean	S.D.	Mean	S.D.	No.	%	No.	%
IV	36	3.9	39.2	2.7	3,365	540	7	19.4	2	5.6
V	109	11.9	39.5	1.9	3,414	523	6	5.5	3	2.8
VI	115	12.5	39.3	2.4	3,339	660	14	12.2	12	10.4
VII	185	20.2	39.1	2.3	3,234	622	25	13.5	22	11.9
VIII	246	26.8	39.1	2.2	3,263	603	29	11.8	24	9.8
IX	226	24.7	39.9	1.7	3,383	503	9	4.0	9	4.0
Subtotal	917	100.0	39.4	2.1	3,318	583	90	9.8	72	7.9
Unknown	100	9.8	39.1	2.2	3,296	615	14	14.0	8	8.0
Total	1,017	100.0	39.4	2.1	3,316	586	104	10.2	80	7.9
Reference	37,134	—	39.4	2.0	3,275	509	3,392	9.1	2,087	5.6
Comparison			*t* = 0.0	*P* = 1.0	*t* = 2.5	*P* = .01	1.1	(0.9–1.4)*	1.4	(1.1–1.8)*
With folic acid	580	57.0	39.5	2.0	3,334	577	51	8.8	42	7.2
Without folic acid	437	43.0	39.2	2.2	3,291	597	53	12.1	38	8.7
Comparison			*t* = 2.3	*P* = .03	*t* = 1.2	*P* = .07	0.7 (0.5–1.0)*		0.8 (0.5–1.3)*	

*OR (95% CI).

**Table 3:** Distribution of gestational age (preterm, term, postterm) and birth weight (low, average, large) groups in pregnant women without PE (as reference) and with PE, in addition in pregnant women with PE with folic acid supplementation.

Gestational age groups	Pregnant women without PE				Pregnant women with PE				Pregnant women with PE + FAS			
	Birth weight (g)				Birth weight (g)				Birth weight (g)			
	No.	%	Mean	S.D.	No.	%	Mean	S.D.	No.	%	Mean	S.D.
−37	3,392	9.1	2,486	435	104	10.2	2,401	460	51	8.8	2,440	401
38–41	29,994	80.8	3,322	428	799	78.6	3,376	491	460	79.3	3,384	506
42−	3,748	10.1	3,612	485	114	11.2	3,729	473	69	11.9	3,663	468
Total	37,134	100.0	3,275	509	1,017	100.0	3,316	580	580	100.0	3,334	57

Birth weight groups	Gestational age (wk)				Gestational age (wk)				Gestational age (wk)			

−2499	2,087	5.6	35.6	3.3	80	7.9	35.7	2.8	42	7.2	36.1	2.8
2500–3999	32,286	87.0	39.5	1.7	826	81.2	39.5	1.7	471	81.2	39.6	1.7
4000−	2,761	7.4	41.0	1.1	111	10.9	41.0	1.2	67	11.6	40.7	1.3
Total	37,134	100.0	39.4	2.0	1,017	100.0	39.4	2.1	580	100.0	39.5	2.0

**Table 4:** The effect of folic acid for birth outcomes of pregnant women with early and late onset PE.

Gestational months	No.	%	Gestational age (wk)		Birth weight (g)		Preterm birth		Low birth weight	
			Mean	S.D.	Mean	S.D.	No.	%	No.	%
All										
IV-VI	260	28.4	39.4	2.2	3,374	589	27	10.4	17	6.5
VII-IX	657	71.6	39.4	2.1	3,296	579	63	9.6	55	8.4
Total	917	100.0	39.4	2.1	3,318	583	90	9.8	72	7.9
with folic acid supplementation										
IV-VI	147	27.2	39.6	2.1	3,419	560	10	6.8	7	4.8
VII-IX	394	72.8	39.4	2.0	3,302	575	38	9.6	32	8.1
Total	541	100.0	39.5	2.0	3,334	573	48	8.9	39	7.2
